# Comparison of CURB-65 and qSOFA scores in predicting outcomes in community-acquired pneumonia

**DOI:** 10.1097/MS9.0000000000004444

**Published:** 2026-01-22

**Authors:** Manoj Gaire, Diwakar Koirala, Arun Gautam, Bivek Mishra, Ramesh Sapkota, Sahil Niraula, Birendra Bhagat, Aayush Adhikari, Aadesh Poudel, Susmin Karki

**Affiliations:** a Department of Emergency Medicine, Devdaha Medical College, Nepal; b Department of Emergency Medicine, B.P. Koirala Institute of Health Sciences, Nepal; c Department of Internal Medicine, B.P. Koirala Institute of Health Sciences, Nepal; d Department of Obstetrics and Gynecology, National Medical College, Birgunj, Nepal; e Department of Emergency Medicine, Manang Hospital, Nepal; f Department of Emergency Medicine, Institute of Medicine, Maharajganj, Nepal

**Keywords:** community-acquired pneumonia (CAP), CURB-65, ICU admission, mortality prediction, qSOFA

## Abstract

**Introduction:**

Community-acquired pneumonia (CAP) is a significant cause of morbidity and mortality worldwide, with high hospitalization and death rates. Early and accurate assessment of disease severity in emergency settings is crucial for guiding treatment and reducing associated costs and mortality. The confusion, urea, respiratory rate, blood pressure, and age ≥ 65 (CURB-65) and quick Sequential Organ Failure Assessment (qSOFA) scoring systems are commonly used tools for prognostication, each with unique strengths. This study aims to compare the predictive capabilities of CURB-65 and qSOFA for intensive care unit (ICU) admission, mortality, and hospital stay duration among CAP patients.

**Methods:**

This cross-sectional, prospective study was conducted over a year at a tertiary hospital. Ninety-three adult CAP patients meeting the inclusion criteria were enrolled. Data on clinical presentation, CURB-65, and qSOFA scores were recorded upon emergency admission. Patients were followed until discharge or death. Statistical analyses, including chi-square tests and area under the receiver operating characteristic (AUROC) curves, were used to compare outcomes predicted by the two scoring systems.

**Discussion:**

Both scoring systems demonstrated utility in predicting adverse outcomes, with qSOFA outperforming CURB-65 for ICU admission (AUROC: 0.817 vs. 0.810). However, CURB-65 was superior in predicting mortality (AUROC: 0.582 vs. 0.535) and hospital stay duration (AUROC: 0.848 vs. 0.829). Positive CURB-65 and qSOFA scores correlated significantly with higher ICU admissions, mortality, and longer hospital stays. Clinical features and comorbidities, such as chronic obstructive pulmonary disease and diabetes, were more prevalent in patients with higher scores.

**Conclusion:**

Both CURB-65 and qSOFA effectively predict ICU admission, mortality, and hospital stay duration in CAP patients. While qSOFA is more sensitive for ICU admission, CURB-65 provides better mortality and length-of-stay predictions, underscoring their complementary roles in clinical decision-making.

## Introduction

Community-acquired pneumonia (CAP) is a leading cause of hospitalization and mortality worldwide, contributing significantly to global health burdens^[^1^]^. When combined with lower respiratory tract infections, CAP ranks as the fourth leading cause of death globally^[^2^]^. It is diagnosed in 5–12% of adults presenting to primary care physicians with symptoms of lower respiratory tract infection^[^3^]^. Even in developed countries, CAP incidence remains high at 9.7 per 1000 persons, with a hospitalization rate of 46.5% and a mortality rate of 12.9%^[^4^]^. In South East Asia, including Nepal, India, Bangladesh, and Indonesia, CAP accounts for 40% of global acute respiratory infections, with 90% of related mortality attributed to bacterial pneumonia^[^5^]^. A cross-sectional study conducted in Nepal by Lamichhane *et al* found pneumonia to be the primary source of infection (45.9%) among 85 patients aged 18–83 years with sepsis and septic shock admitted to a tertiary hospital emergency department over 5 months in 2018^[^6^]^. Despite advances in antimicrobial therapy and life-support measures, CAP remains a significant infectious disease burden, frequently complicated by sepsis and respiratory failure, with mortality rates reaching up to 30%^[^3,7^]^.

HIGHLIGHTS

- Confusion, urea, respiratory rate, blood pressure, and age ≥ 65 (CURB-65) outperforms quick Sequential Organ Failure Assessment (qSOFA) in predicting mortality and hospital stay in community-acquired pneumonia patients.
- qSOFA is superior for predicting intensive care unit admission.
- Both scores are complementary for clinical decision-making in emergency settings.
- Study limitations include a single-center design and a small sample size for high-risk groups.

To guide clinical management, several severity assessment tools have been developed, with CURB-65 and the Pneumonia Severity Index (PSI) being widely validated for CAP prognosis and treatment decisions^[^8,9^]^. The CURB-65 score, endorsed by the British Thoracic Society in 2003, comprises five parameters: confusion, blood urea >7 mmol/L, respiratory rate ≥30/min, systolic blood pressure <90 mmHg and/or diastolic blood pressure ≤60 mmHg, and age ≥65 years, each contributing one point to a score ranging from 0 to 5^[^10^]^. Higher scores correlate with increased mortality, guiding site-of-care decisions. In contrast, the quick Sequential Organ Failure Assessment (qSOFA), introduced by the Third International Consensus Definitions for Sepsis in 2016, is designed to rapidly identify patients at risk of sepsis outside the intensive care unit (ICU)^[^11^]^. qSOFA includes three parameters: low blood pressure (systolic ≤100 mmHg), high respiratory rate (≥22 breaths/min), and altered mentation (Glasgow Coma Scale <15), with scores ranging from 0 to 3. A qSOFA score ≥2 is associated with a higher risk of death or prolonged ICU stay. Unlike the PSI, which requires 20 variables and extensive laboratory workup, or the full SOFA score, which is similarly complex, CURB-65 and qSOFA are simpler, requiring minimal or no laboratory tests^[^11,12^]^.

The use of CURB-65 and qSOFA facilitates early risk stratification in emergency departments, enabling rapid prediction of complications such as mortality, ICU admission, and prolonged hospital stays. These tools support triaging patients, determining appropriate sites of care (e.g., ward vs. ICU), and guiding early referral, particularly in resource-limited settings where access to advanced diagnostics is restricted. qSOFA requires no laboratory investigations, while CURB-65 relies solely on blood urea, reducing economic and time burdens in busy emergency settings. By accurately assessing CAP severity at initial presentation, these scores help clinicians optimize treatment, reduce costs, and minimize mortality, particularly for healthcare providers working in remote or poorly equipped facilities^[^10,11^]^.

This study aims to compare the predictive performance of CURB-65 and qSOFA for outcomes including ICU admission, mortality, and duration of hospitalization in adult CAP patients presenting to the emergency department of a tertiary hospital. By evaluating these tools in a resource-constrained setting like Nepal, this research seeks to guide clinicians in selecting the most appropriate prognostic score for rapid triage and referral, ultimately aiming to reduce the morbidity and mortality associated with CAP^[^10,11^]^.

## Methods

This prospective cross-sectional quantitative study was conducted at a tertiary-level hospital over a period of 12 months. A total of 93 patients diagnosed with CAP were enrolled using a non-probability convenience sampling method. The study site was selected because CAP represents one of the most common clinical presentations requiring emergency evaluation and inpatient care in this hospital. Ethical approval was obtained from the Institutional Review Committee prior to data collection.

The sample size was calculated using the standard formula:

*n* = *Z*^2^ × *P*(1 − *P*)/*e*^2^,

where *Z* = 1.96 for a 95% confidence interval, *P* = 0.04 representing the 4% prevalence of adult CAP^[^13^]^, and *e* = 0.04 as the allowable error. Substituting these values yielded a minimum sample size of 92.198, which was rounded to 93.

All patients aged 18 years and above presenting to the emergency department with features of CAP were evaluated for eligibility. Patients were excluded if they were younger than 18 years, had a fever due to another confirmed cause, had been hospitalized within the preceding 90 days, or were receiving steroids, immunosuppressants, or chemotherapy. Patients with a history of organ transplantation, malignancy, HIV, bronchiectasis, or active or newly diagnosed tuberculosis were also excluded. CAP diagnosis followed the *New England Journal of Medicine* criteria, requiring (1) clinical evidence of infection such as fever, chills, or leukocytosis, (2) respiratory symptoms including cough, shortness of breath, sputum production, chest pain, or extrapulmonary abnormalities, and (3) a new or progressive infiltrate on chest radiography.

**Figure 1.**
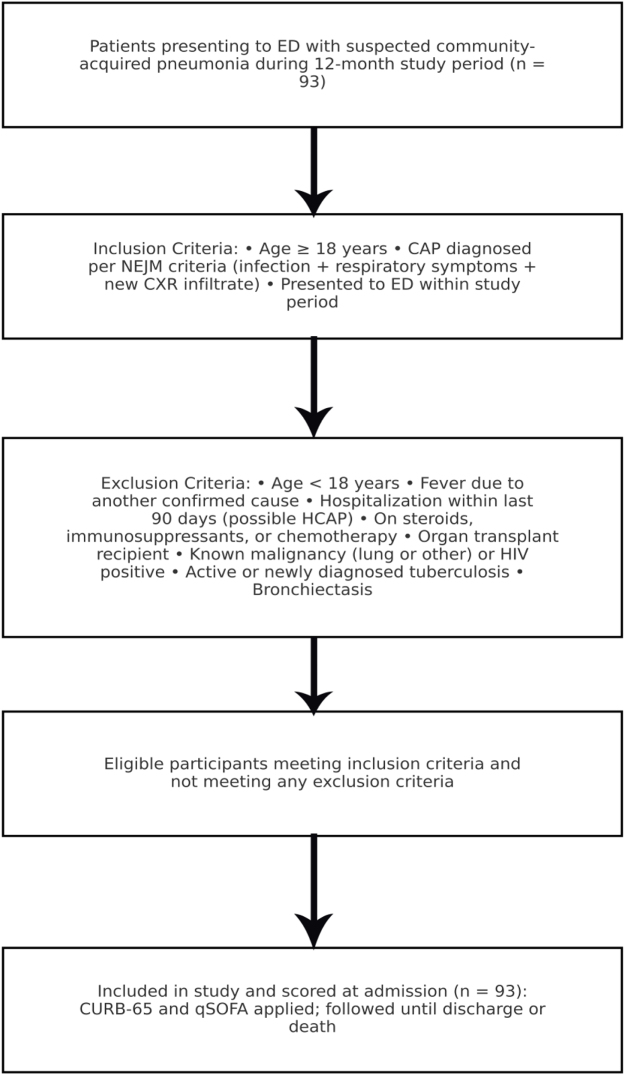
Patient recruitment flowchart.

Upon presentation, detailed history, physical examination findings, demographic characteristics, physiological parameters, and comorbidities were recorded in the emergency department. The CURB-65 and qSOFA scoring systems were applied to each patient, with all score components documented in the pre-designed proforma. Decisions regarding further investigations, supportive management, admission to ward or ICU, or discharge were made by the treating physician in accordance with hospital emergency protocols. Each patient was followed until discharge or in-hospital death. The patient recruitment is described in Figure 1.

Data were entered into Microsoft Excel and analyzed using SPSS (Statistical Package for the Social Sciences) version 21. Continuous variables were summarized as mean ± standard deviation (SD) and analyzed using the independent *t*-test or one-way ANOVA, where appropriate. Categorical variables were expressed as frequencies and percentages, with associations assessed using the Chi-square test. Receiver operating characteristic (ROC) curves and the area under the curve were employed to compare the predictive accuracy of confusion, urea, respiratory rate, blood pressure, and age ≥ 65 (CURB-65) and qSOFA scores for outcomes including ICU admission, mortality, and duration of hospital stay. All statistical analyses were two-tailed, and a *P*-value <0.05 was considered statistically significant. Results are presented in tables and graphs.

## Ethics

The reporting of the case has been done according to the Helsinki Declaration of 1975. The ethical standards are in accordance with the guidelines provided by the Committee for the Purpose of Control and Supervision of Experiments on Animals (CPCSEA) and the Indian Council of Medical Research (ICMR).

## Results

In the present hospital-based cross-sectional study, which enrolled 93 patients, the mean age of the participants was 57.87 (SD ±18.13) with a minimum age of 18 years and a maximum age of 85 years. The mean duration of hospital stay was 7.10 days (SD ±3.74), with a maximum hospital stay duration of 18 days and a minimum of 1 day. The hospitalization rate was 88.17 %. The findings of this study are interpreted below in the form of graphs and tables.

**Figure 2.**
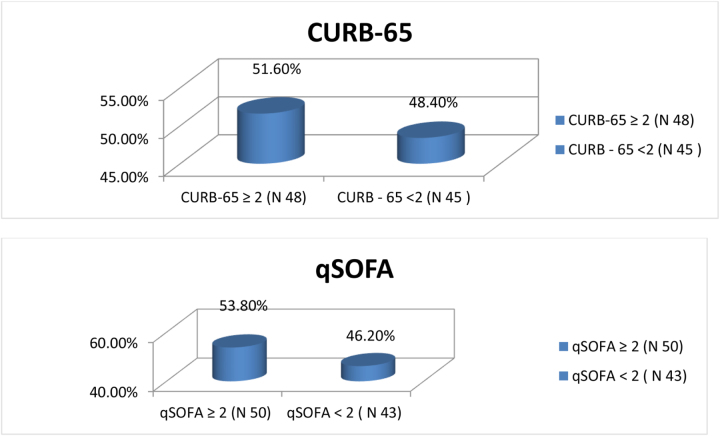
Frequency of participants with positive CURB-65 (CURB-65 ≥ 2) and negative CURB-65 (CURB-65 < 2), and positive qSOFA (qSOFA ≥ 2) with negative qSOFA (qSOFA < 2).

Frequency, mortality, and ICU admission of patients at different CURB-65 scores are given in Table 1. Similarly, Figure 2 shows CURB-65 and qSOFA admission in a bar graph. Patients with a CURB-65 score of 0 were 11 in number with no ICU admission and mortality and those with a score of 1 were 34 in number with 8.8% ICU admission and 10 % mortality. Similarly, patients with scores of 2, 3, 4, and 5 have patient frequency of 22, 20, 3, and 3 with ICU admission rates of 45.4%, 90%, 66.6%, and 100% and mortality of 20%, 20%, 20%, and 30%, respectively. Similarly, patients with a CURB-65 score of 5 have the highest percentage of ICU admission (100%) and the lowest in CURB 65 score of 0 (0%).

**Table 1:** Frequency of participants at various CURB-65 scores with ICU admission, mortality, and duration of hospital stay

CURB-65 score	Frequency (*n* = 93)	ICU admission (*n* = 36)	Mortality (*n* = 10)	Duration (days), mean (SD)
0	11	0 (0%)	0 (0%)	2.55 (±2.42)
1	34	3 (8.8%)	1 (10%)	5.68 (±3.03)
2	22	10 (45.4%)	2 (20%)	8.09 (±2.26)
3	20	18 (90%)	2 (20%)	9.75 (±2.71)
4	3	2 (66.6%)	2 (20%)	13.33 (±4.16)
5	3	3 (100%)	3 (30%)	8.67 (±5.55)

Patients with a qSOFA score of 0 were 4 in number with no ICU admission and no mortality and those with a score of 1 were 41 in number with 7.3 % ICU admission and 40% mortality. Similarly, patients with scores of 2 and 3 have patient frequency of 40 and 8, with 65% ICU admission and 87.5% ICU admission and 0 mortality and 60% mortality, respectively. Similarly, patients with a qSOFA score of 3 have the highest percentage of ICU admission (87.5%) and 60% of mortality and the lowest in a qSOFA score of 0 (0%)

Figure 4 shows that out of the 93 patients, the number of patients in positive CURB-65 was 48 (51.6%) and negative CURB-65 was 45 (48.4%). Similarly, the number of patients in positive qSOFA was 50 (53.8%) and negative qSOFA (qSOFA: 2) was 43 (46.20%).

Table 2 shows that fever was present in 48 patients with positive CURB-65 and cough with sputum production was present in 44 patients. Similarly dyspnea, confusion, and diarrhea were present in 48, 12, and 11 patients, respectively. Two patients had other clinical features.

**Table 2:** Clinical presentation of patients with positive and negative CURB-65 scores

Clinical features	CURB-65 ≥ 2 (positive CURB-65, *n* = 48)	CURB < 2 (negative CURB-65, *n* = 45)	*P*-value
Fever (*n* = 88)	48 (100%)	40 (88.8%)	0.018
Cough with sputum (*n* = 77)	44 (91.6%)	33 (73.3%)	0.019
Dyspnea (*n* = 87)	48 (100%)	39 (86.6%)	0.009
Confusion (*n* = 13)	12 (25%)	1 (2.2%)	0.002
Diarrhea (*n* = 15)	11 (22.9%)	4 (8.8%)	0.066
Other (*n* = 4)	2 (4.1%)	2 (4.4%)	0.947
	qSOFA ≥ 2 (positive qSOFA), *n* = 50	qSOFA < 2 (negative qSOFA), *n* = 43	
Fever (*n* = 88)	50 (100%)	38 (88.8%)	
Cough with sputum (*n* = 77)	47 (94%)	30 (69.7%)	
Dyspnea (*n* = 87)	49 (98%)	38 (88.3%)	
Confusion (*n* = 13)	12 (24%)	1 (2.3%)	
Diarrhea (*n* = 15)	10 (20%)	5 (11.6%)	
Other (*n* = 4)	4 (8%)	0 (0%)	
	Positive CURB-65 (CURB ≥ 2), *n* = 48	Negative CURB-65 (CURB < 2), *n* = 45	
Diabetes mellitus (*n* = 22)	Present	17 (35.4%)	5 (11.1%)
Hypertension (*n* = 18)	Hypertensive	9 (18.75%)	9 (20%)
COPD (*n* = 34)	Present	24 (50%)	10 (22.2%)
Alcohol (*n* = 22)	Consumer	10 (20.8%)	12 (26.6%)
Past history of PTB (*n* = 14)	Present	12 (25%)	2 (4.4%)
Smoking (*n* = 27)	Smoker	14 (29.1%)	13(28.8%)
	Positive qSOFA (QSOFA ≥ 2) *n* = 50	Negative qSOFA (QSOFA < 2), *n* = 43	
Diabetes mellitus (*n* = 22)	Present	18 (36%)	4 (9.3%)
Hypertension (*n* = 18)	Hypertensive	7 (14%)	11 (25.5%)
COPD (*n* = 34)	Present	25 (50%)	9 (20.9%)
Alcohol (*n* = 22)	Consumer	10 (20%)	12 (27.9%)
Past history of PTB (*n* = 14)	Present	12 (24%)	2 (4.6%)
Smoking (*n* = 27)	Smoker	13 (26%)	14 (32.5%)

Similarly in negative CURB-65, out of 45 patients fever was present in 40 patients. Cough with sputum production was present in 33 patients. Similarly, dyspnea, confusion, and diarrhea were present in 39, 1, and 4 patients, respectively. Two patients had other clinical features. Fever was present in 50 out of 50 patients with positive qSOFA, and cough with sputum production was present in 47 patients. Similarly dyspnea, confusion, and diarrhea were present in 49, 12, and 10 patients, respectively. Four patients had other clinical features.

Similarly in negative qSOFA, out of 43 patients fever was present in 38 patients. Cough with sputum production was present in 30 patients. Similarly dyspnea, confusion, and diarrhea were present in 38, 1, and 5 patients, respectively. There were no other clinical features in patients with negative qSOFA. Diabetes was present in 17 out of 48 patients with positive CURB-65 score. Similarly, other co-morbidities like hypertension (HTN), chronic obstructive pulmonary disease (COPD), and past history of pulmonary tuberculosis (PTB) were associated with 9, 24, and 12 patients, respectively. About 10 patients consume alcohol and 14 patients were smoker. Diabetes was present in 5 patients out of 45 patients in negative CURB-65; similarly, other co-morbidities like HTN, COPD, and past history of PTB were present with 9, 10, and 2 patients, respectively. About 12 patients consume alcohol and 13 patients were smoker. Diabetes was present in 18 patients out of 50 in positive qSOFA; similarly, other co-morbidities like HTN, COPD, and past history of PTB were present in 7, 25, and 12 patients, respectively. About 10 patients consume alcohol and 13 patients were smoker.

Diabetes was present in 4 patients out of 43 in negative CURB-65; similarly, other co morbidities like HTN, COPD, and past history of PTB were associated with 11, 9, and 2 patients, respectively. About 12 patients consume alcohol and 14 patients were smoker.

Table 3 shows patient with positive CURB-65, 9 patients had blood urea level more than 7 mmol/L and 39 patients had urea less than 7; similarly, in negative CURB-65 there were no patient having urea more than 7 mmol/L but 45 patients had urea less than 7.

**Table 3:** CURB-65 and urea relation

CURB-65	Urea > 7 mmol/L	Urea <7 mmol/L
Positive CURB-65 (*n* = 48)	9 (18.8%)	39 (81.3%)
Negative CURB-65(*n* = 45)	0	45 (100%)

Out of the 93 patients, blood culture was done in 42 (45.1 %) patients from which 7 (16.6 %) patients had positive culture and no organisms were found in 35 patients. Most common organism isolated was *Streptococcus pneumoniae* in four (57 %) patients, *Klebsiella* in one (14.2%) patient, *Legionella* in one (14.2%) patient, and *Hemophilus influenza in* one(14.2 %) patient.

## Outcomes

Table 4 shows that among patients with a positive CURB-65 score, 33 were admitted to ICU and 15 were admitted to ward, whereas among patients with a negative CURB-65 score, 3 were admitted to ICU, 31 in ward, and 11 were discharged from emergency.

**Table 4:** Site of treatment

	ICU admission	Ward admission	Discharge from ER	*P*-value for ICU admission
CURB-65				
CURB-65 ≥ 2 (*n* = 48)	33 (68.7%)	15 (31.25%)	0 (0%)	
CURB-65 < 2 (*n* = 45)	3 (6.6%)	31 (68.8%)	11 (24.4%)	<0.001
qSOFA				
qSOFA ≥ 2 (*n* = 50)	34 (68%)	16 (32%)	0 (0%)	
qSOFA < 2 (*n* = 43)	2 (4.6%)	30 (69.7%)	11 (25.5%)	<0.001

In positive qSOFA score, 34 were admitted to ICU and 16 were admitted to ward, whereas in negative qSOFA, 2, 30, and 11 patients were admitted to ICU, ward, and discharge from emergency, respectively.

**Figure 3.**
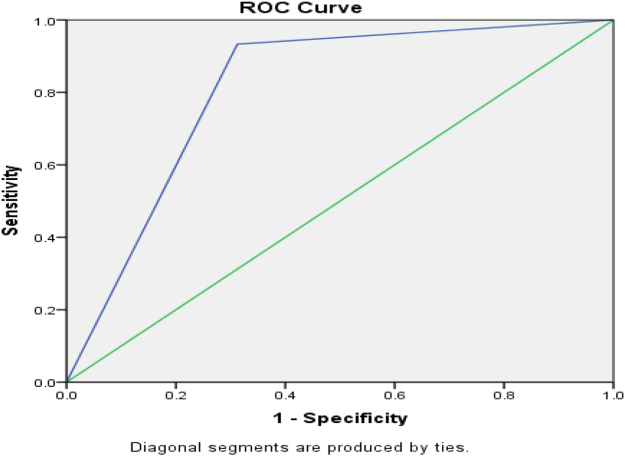
AUROC curve of positive CURB-65 = 0.810 (96 % CI) for ICU admission.

**Figure 4.**
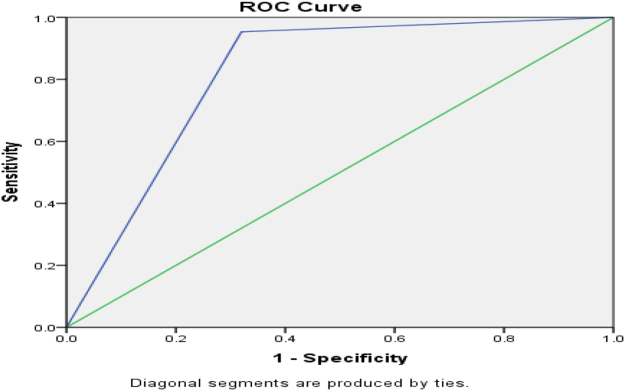
AUROC curve of positive qSOFA = 0.817 (95 % CI) for ICU admission.

For ICU admission, sensitivity and specificity of both positive CURB-65 and positive qSOFA are as follows:

Figure 3 shows AUROC for ICU admission for CURB-65, and Figure 4 shows the AUROC curve for qSOFA for ICU admission.

Sensitivity and specificity of positive CURB-65 and positive qSOFA in relation to mortality are as follows:

Table 5 shows that out of the 48 patients with a positive CURB-65 score, death occurred in 9 patients and the remaining 39 improved. Similarly out of the 45 patients in negative CURB-65, 1 patient died and the remaining 44 were improved.

**Table 5:** Mortality

	Expired (*n* = 10)	Improved (*n* = 83)	*P*-value for expired
CURB-65			
CURB ≥ 2 (*n* = 48)	9 (18.75%)	39 (81.25%)	0.01
CURB < 2 (*n* = 45)	1 (2.22%)	44 (97.77%)	
qSOFA			
qSOFA ≥ 2 (*n* = 50)	7 (14%)	43 (86%)	0.01
qSOFA < 2 (*n* = 43)	3 (6.97%)	40 (93.02%)	

Out of the 50 patients with positive qSOFA, death occurred in 7 patients and the remaining 43 were improved. Similarly out of the 45 patients in negative qSOFA, 3 patient died and the remaining 40 were improved.

**Figure 5.**
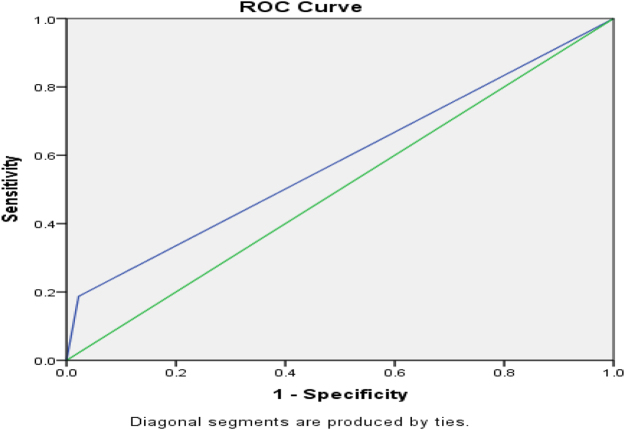
The AUROC curve of positive CURB ≥ 2 is 0.582 (95 % CI) for hospital mortality.

**Figure 6.**
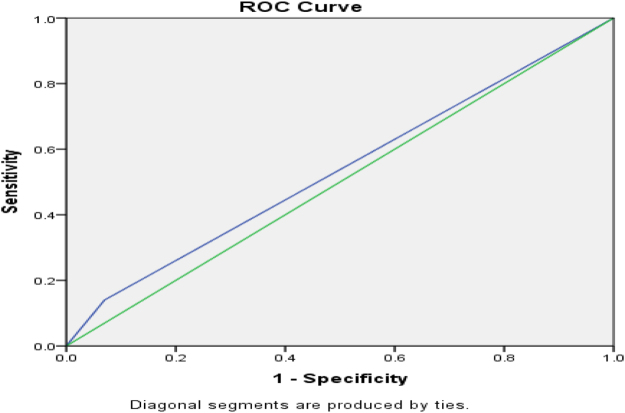
The AUROC curve of positive qSOFA ≥ 2 is 0.535 (95% CI) for hospital mortality.

Looking upon the AUROC curve between positive CURB-65 and positive qSOFA, AUROC of positive CURB-65 is 0.582 and positive qSOFA is 0.535, as shown in Figures 5 and 6.

Table 6 shows that the mean duration of hospital stay was 9.15 days (SD ±3.018) in patients with positive CURB and 4.92 days (SD ±3.175) in patients with negative CURB-65. Similarly, the mean duration of hospital stay was 8.90 days (SD ±3.018) in patients with positive qSOFA and 5.00 days (SD ±3.50) in patients with negative qSOFA.

**Table 6:** Duration of hospital stay

Scores	Duration (days), mean (SD)	*P*-value
CURB-65 ≥ 2	9.15 (±3.018)	
CURB-65 < 2	4.92 (±3.175)	<0.001
qSOFA ≥ 2	8.90 (±3.018)	
qSOFA < 2	5.00 (±3.50)	<0.001

**Figure 7.**
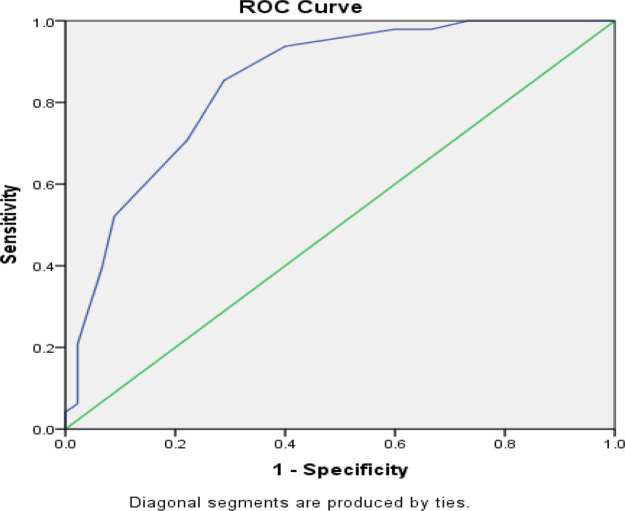
The AUROC curve of positive CURB-65 ≥ 2 is 0.848 (95 % CI) for the duration of hospital stay.

**Figure 8.**
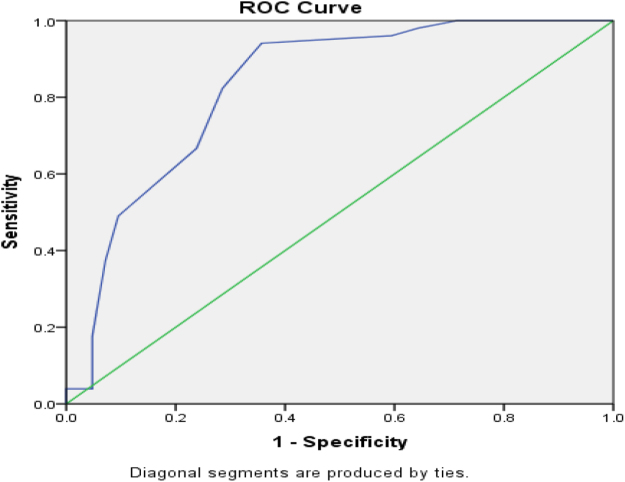
The AUROC curve of positive qSOFA ≥ 2 is 0.829 (95 % CI) for the duration of hospital stay.

Looking upon the AUROC curve of the hospital stay duration of both prognostic scoring systems, the AUROC curve of CURB-65 ≥ 2 is 0.848 (95 % CI) and the AUROC curve of qSOFA ≥ 2 is 0.829 (95% CI), as shown in Figures 7 and 8.

The work has been reported in line with the STROCSS criteria^[^14^]^.

## Discussion

CURB-65 is simple and pragmatic, relying on five readily measurable variables; urea is the only laboratory investigation required to stage severity, making the score feasible in primary and secondary care. It may also aid community physicians in triage decisions when blood urea is unavailable^[^10^]^.

The 2016 sepsis guidelines state that adult ED or ward patients with suspected infection are at higher risk of adverse outcomes if they meet ≥2 qSOFA criteria – respiratory rate ≥22/min, altered mentation, or systolic blood pressure ≤100 mmHg – and such patients warrant prompt treatment and consideration for ICU^[^11^]^. Evidence is mixed: Singer *et al* reported that qSOFA predicts ICU admission, inpatient mortality, and length of stay in adults likely to be admitted, with or without suspected infection^[^15^]^; Müller *et al* found qSOFA superior to CURB-65 for prognosticating ICU admission^[^16^]^; in contrast, Grudzinska *et al* observed poorer performance of qSOFA than the CAP-specific CURB-65 for early identification of patients at risk of ICU admission or death, supporting organ-specific tools^[^17^]^; Finkelsztein *et al* noted higher in-hospital mortality among qSOFA-positive (≥2) than qSOFA-negative patients^[^18^]^; Ranzani *et al* concluded CURB-65 outperforms qSOFA for mortality prediction^[^19^]^; and Tokioka *et al* found qSOFA inferior to CURB-65 for 28-day mortality but similar for ICU admission^[^20^]^. In our cohort, 49/93 (52.7%) were male and 44/93 (47.3%) female; the largest age group was 66–75 years (29/93, 31.2%), followed by 56–65 years (24/93, 25.8%), with incidence rising with age – consistent with Almirall *et al* and Shah *et al*^[^21,22^]^ – and male predominance likely reflecting higher exposure to risk factors such as smoking and COPD. The mean age among deaths was 72.2 years versus 56.07 years among survivors, underscoring advanced age as a predictor of mortality, in line with Luna *et al*, Ito *et al*, and Koivula *et al*^[^23–25^]^. Clinically, fever occurred in 88/93 (94.4%), productive cough in 77/93 (82.8%), and dyspnea in 87/93 (93.5%), followed by diarrhea 14/93 (16.1%) and confusion 13/93 (14%); all had new consolidation on chest radiography – similar to Hageman *et al*, Müller *et al*, and Shrestha *et al*^[^16,26,27^]^.

Comorbidity burden was high (74/93, 79.5%): COPD (36.6%) was most frequent, then smoking (29%), diabetes (23.7%), and alcohol use (23.7%), mirroring Koivula *et al*, Molinos *et al*, Müllerová *et al*, and Rosón *et al*^[^24,28–30^]^. The prominence of COPD likely reflects age, smoking prevalence, and inhaled steroid exposure; diabetes and alcohol use further impair host defenses, and smoking cessation remains central to CAP prevention. Symptom frequencies were higher in positive CURB-65 (≥2) and qSOFA (≥2); fever was universal in positive groups and statistically significant versus negative scores (*P* = 0.018), as were productive cough, dyspnea, and confusion, whereas diarrhea was not (*P* = 0.06), consistent with gastrointestinal symptoms occurring in up to 20% of CAP cases^[^12^]^. Positive-score groups also had more comorbidities – particularly diabetes, COPD, and prior PTB – with significant associations. Positive sputum and blood cultures were more common with CURB-65 ≥ 2 and qSOFA ≥ 2 (*P* < 0.001), indicating greater microbiological yield with severity, similar to Müller *et al*^[^16^]^.

ICU admission was frequent in positive strata: 33/48 (68.75%) for CURB-65 ≥ 2 and 34/50 (68%) for qSOFA ≥ 2; admission rates rose with score and were significant versus negative scores (*P* < 0.01). For predicting ICU admission, sensitivity/specificity were 91.7%/73.7% for CURB-65 ≥ 2 and 94.4%/71.9% for qSOFA ≥ 2; AUROC was 0.810 (95% CI) for CURB-65 and 0.817 (95% CI) for qSOFA, suggesting marginally better discrimination by qSOFA, potentially due to its lower respiratory-rate threshold (≥22 vs. ≥30). These findings align with Müller *et al*^[^16^]^, but differ from Tokioka *et al*^[^20^]^, likely reflecting center-specific ICU criteria and hospitalization thresholds^[^17,21^]^. Mortality rose with score: 9/48 (18.75%) deaths with CURB-65 ≥ 2 and 7/50 (14%) with qSOFA ≥ 2 (both *P* < 0.01 vs. negative).

For in-hospital mortality, CURB-65 ≥ 2 had higher sensitivity/specificity (90%/53%) than qSOFA ≥ 2 (70%/48.2%); AUROC was 0.582 (95% CI) for CURB-65 versus 0.535 (95% CI) for qSOFA, indicating better mortality prediction by CURB-65, plausibly due to inclusion of urea and age – CAP-relevant prognosticators – consistent with Ranzani *et al* and Tokioka *et al*^[^19,20^]^. Finally, length of stay increased with severity: CURB-65 ≥ 2, 9.15 ± 3.01 days versus <2, 4.91 ± 3.17 days; qSOFA ≥ 2, 8.90 ± 2.92 days versus <2, 5.00 ± 3.50 days (both *P* < 0.01), with AUROC 0.84 (95% CI) for CURB-65 and 0.82 (95% CI) for qSOFA, suggesting a slight advantage for CURB-65 in predicting prolonged hospitalization.

## Conclusions

Both CURB-65 and qSOFA can identify patients with CAP for ICU admission, hospital mortality, and duration of hospital stay. CURB-65 was better in predicting both hospital mortality and duration of stay; in contrast, qSOFA was better at identifying patients for ICU admission. ICU admission, mortality, and duration of hospital stay progressively increase with increasing scores in both prognostic scoring systems.

## Data Availability

No data sheets were used to analyze the research.
